# Policy Literacy, Barriers, and Gender Impact on Accessibility to Healthcare Services under Compulsory Migrant Health Insurance among Myanmar Migrant Workers in Thailand

**DOI:** 10.1155/2020/8165492

**Published:** 2020-12-29

**Authors:** Sa Hlyan Htet Naing, Sang-Arun Isaramalai, Phen Sukmag

**Affiliations:** ^1^Health Impact Assessment Research Center, Health System Management Institute, Prince of Songkla University, Songkhla 90110, Thailand; ^2^Faculty of Nursing, Prince of Songkla University, Songkhla 90110, Thailand

## Abstract

Accessibility to health service and experience of healthcare are important factors for public health policymaking. The current study aimed to describe the status of accessibility and barriers to getting care as well as policy literacy among Myanmar migrant workers and ultimately to identify the predictors of accessibility to healthcare services among this population through Thailand's Compulsory Migrant Health Insurance (CMHI). A cross-sectional survey was used to collect data from 240 Myanmar migrant workers who were 18 years or older, resided in Songkhla Province, and had Compulsory Migrant Health Insurance. The instrument was a set of questionnaires consisting of a Personal Data Form, Policy Literacy Questionnaire, Barriers to Get Care Questionnaire, and Accessibility to Healthcare Services Questionnaire. Descriptive statistics, correlation analysis, and multiple regression analysis were used to analyze data. The majority of participants had a high level of policy literacy (36.3%), barriers to get care (34.2%), and accessibility to health care services (35.8%). Policy literacy (*β* = 0.35, *p* < 0.001), barriers to get care (*β* = −0.32, *p* < 0.001), and gender (*p* < 0.001) were significant predictors of accessibility to healthcare services and could explain 43.2% of the total variance. To increase the accessibility to healthcare services among migrant workers with Compulsory Migrant Health Insurance, public health policymakers are recommended to cooperate more with healthcare staff and the workers' employers to enhance the distribution of information about the health insurance to decrease barriers to get care.

## 1. Introduction

Due to its rapid economic growth, Thailand has become a popular destination country for migrant workers. Thailand's 2019 migration report estimated nearly 3.9 million migrant workers from neighboring countries including Myanmar, Laos, and Cambodia are working in Thailand [1]. Myanmar migrant workers have been officially encouraged to work in Thailand under the 2002 Thai-Myanmar agreement [2]. These workers are usually employed in factories, restaurants, construction sites, shops, and small businesses as well as in the agricultural sector or domestic service. Besides, migrant laborers, especially in low-skilled sectors, have health risks due to poor working conditions, lack of health insurance, and lack of safety standards among the employers [3].

To look after the health of migrant workers, the Thai government provides two types of health insurance: Social Security Scheme and Compulsory Migrant Health Insurance (CMHI) scheme. The Social Security Scheme is limited to migrants who have valid work permits, whereas the CMHI scheme serves both migrants and their dependents [2, 4]. Under the CMHI scheme, eligible members can receive an annual check-up and other health services [5]. Unfortunately, obtaining insurance does not always guarantee access to care. Frenk and White (1992) defined the term “access” as the “ability of the population to seek and obtain care.” Although accessibility is a basic outcome of healthcare services in many countries, migrants are excluded from the well-established Thai public health system [5]. Sustainable public health policies that promise fair access to migrant workers require policymakers to understand accessibility and migrants' experience of accessibility to a health service. Therefore, an appropriate assessment of migrant workers' ability to get the care and the provision of care are essential to fill the knowledge gap for the Thai policymakers.

Moreover, improving the health status of migrant populations is beneficial to the host country's health security and economy. Promoting the health status of migrants and their accessibility to health care services is thus significant to Thailand. This study aimed to describe the status of ability and barriers to getting care as well as policy literacy among Myanmar migrant workers with CMHI. The study ultimately aimed to identify the predictors of the accessibility to healthcare services among this population. The study results provide a better understanding of the accessibility to healthcare services by Myanmar migrant workers along with its predictors and guidance to design proper interventions and policies to promote their health.

## 2. Organizing Framework of Study

The theoretical framework of the accessibility to healthcare services was based on the framework of access to health services developed by Peter et al. [6], which was based on four dimensions: geographic accessibility, availability, financial accessibility, and acceptability of services. Geographic accessibility refers to the distance and travel time to access healthcare services. Availability refers to the available healthcare services, providers, and medicines. Financial accessibility refers to the cost of healthcare access. Acceptability refers to the patients' satisfaction of provided healthcare services from the CMHI scheme.

For this study, the factors considered to be determinants of the accessibility to healthcare services were based on the findings of previous studies. Individual characteristics, policy literacy, and barriers to getting care are viewed as predictors of the accessibility to healthcare services. Among individual characteristics, gender, education, marital status, income, and health status have also been shown to be associated with the accessibility to healthcare services of migrant workers in Thailand [7–9].

The concept of policy literacy in this study was based on the literature and evidence of health literacy, which seems to be a part of policy literacy [10, 11]. Kwan et al. (2006) defined health literacy as “the degree to which people can access, understand, appraise, and communicate information to engage with the demands of different health contexts to promote and maintain good health across the life-course” [10]. Studies have shown that health literacy to be associated with access to health services [12]; individuals with limited health literacy were significantly more likely to delay seeking healthcare [13]. Therefore, policy literacy in this study included the ability to find, understand, appraise, and communicate information on policy related to a health insurance scheme.

Barriers to getting care in this study included personal barriers, financial barriers, social barriers, health system barriers, healthcare provider barriers, and work situation barriers which were modified from two studies [14, 15]. Barriers such as financial difficulty, location of services, lack of support from family and friends [16], attitudes among healthcare personnel [17], communication difficulties, perceived social marginalization [18], and cultural barriers have all been shown to significantly influence access to healthcare services. Reducing these barriers may increase an individual's ability to access healthcare [19].

This study aimed to explore the policy literacy, barriers to getting care and ability to get the care and to examine the predictability of policy literacy, barriers, and individual characteristics such as gender, education, marital status, income, and health status on the accessibility to healthcare services among Myanmar migrant workers living in Songkhla Province of Southern Thailand.

## 3. Materials and Methods

### 3.1. Study Design

A cross-sectional design was used for this study.

### 3.2. Setting and Samples

The study was conducted in Songkhla Province which is one of the major cities in Southern Thailand. Due to its vibrant economy, the city attracts many migrant workers from various ethnic groups, beliefs, and cultures. The study population was comprised of Myanmar migrant workers who had Compulsory Migrant Health Insurance. The required sample size was calculated based on the formula proposed by Green (1991) for regression analysis. The estimated sample size required for this study was 111 while 7 predictors were used. However, to obtain an equal distribution number of two groups of migrant workers, those who had experience using the service and no experience on using the service, 9 more subjects were added to be 120 subjects then proceeded to double the sample size to gain more generalizability. Overall, 240 migrant workers were selected from the four main sectors of employment representative of such population including agriculture, construction, seafood processing, and services. The sampling process consisted of two steps. First, the migrant workers' residence or workplace was located by purposive sampling. This method was used because migrant workers in these occupations are widespread hence hard to reach. Second, after selecting the locations, quota sampling was used to represent the population as much as possible by distributing the Myanmar migrant workers according to their experience with health services, occupational status, and gender.

### 3.3. Data Collection

Data were collected between March and May of 2019 and the interviews were conducted at the locations of work or residences depending on the convenience or preference of the workers. The questionnaires were distributed to the literate migrant workers and questions were explained by the interviewer. For those who were unable to read or write the Myanmar language, the interviewer completed the questionnaire on their behalf via a face-to-face interview.

### 3.4. Ethical Considerations

Permission was obtained from the Institutional Review Board of Health System Management Institute from Prince of Songkla University, Thailand. Informed individual consent was obtained from all participants after explaining that their participation was entirely voluntary. Moreover, the participants were assured that their responses would be kept strictly confidential while their anonymity guaranteed.

### 3.5. Measurements

A questionnaire with four sections, personal characteristics, policy literacy, barriers to getting care, and the accessibility to healthcare services, was designed for the study. Twelve items of personal characteristics were collected using a separate questionnaire designed by the researchers.

The questionnaire of policy literacy was developed by the researchers based on previous studies on health literacy since there is no instrument ever developed for policy literacy [5, 10, 11]. The policy literacy questionnaire comprised of 33 items, 20 of which were positively worded while the other 13 items were negatively worded. These items were separated into four subscales: the ability to find information about the CMHI scheme, the ability to understand the information, the ability to appraise the information, and the ability to communicate this information to others. Responses for each item were scored on a 4-point Likert scale, ranging from 1 to 4, where 1 = strongly disagree, 2 = disagree, 3 = agree, and 4 = strongly agree. Responses to the negatively worded items were reversed in order before summing the total scores. Higher scores indicated a higher level of policy literacy. Possible total scores for the scale ranged from 33 to 132. The reliability of the questionnaire was assessed by testing the questionnaire on 30 Myanmar migrant workers who were not included in the main study which resulted in a Cronbach's alpha of 0.85, indicating a high degree of reliability.

Barriers to getting care were measured using a 28-item questionnaire developed by the researchers based on previous studies investigating barriers to access healthcare [14–16, 20, 21]. The questionnaire contained six subscales: personal barriers, financial barriers, social barriers, healthcare provider barriers, health system barriers, and work situation barriers. The responses for all items were recorded on a 4-point Likert scale that ranged from 1 (strongly disagree) to 4 (strongly agree). All items were negatively worded questions. Total scores ranged from 28 to 112. Cronbach's alpha of the questionnaire was 0.90.

The questionnaire measuring accessibility to healthcare services consisted of four parts, geographic accessibility, availability, financial accessibility, and acceptability, and also included 24 items, 18 positive and 6 negative items. The questionnaire was also developed by the researchers based on previous studies on accessibility to get care and information provided by the CMHI scheme [5, 6, 22]. Like the other questionnaires, the responses were assessed on a 4-point Likert scale ranging from 1 (strongly disagree) to 4 (strongly agree), while the scores for negative items were rated from 4 (strongly disagree) to 1 (strongly agree). Higher scores indicated a higher level of perceived accessibility to healthcare services. The total score for the accessibility to healthcare services ranged between 24 and 96. For this questionnaire, Cronbach's alpha was 0.91.

The English version of the questionnaire was examined for content validity by a panel of three experts in areas of community health, health system, and public health. Modifications were made on each expert's recommendations. The questionnaires were translated into Myanmar and then into English again using back translation technique by two bilingual speakers. The differences of two English versions (original vs. back translated) were discussed with those two translators. Finally, the Myanmar version was modified based on agreement of the translators to maintain language congruency with the original version approved by those three experts.

### 3.6. Data Analysis

Data analysis was conducted using SPSS 17 for Windows. Descriptive statistics such as frequencies, percentages, means, and standard deviations were used to describe personal data, policy literacy, barriers to getting care, and the accessibility to healthcare services. For a better understanding of the policy literacy scores, barriers to getting care, and the accessibility to healthcare services, all scores including subdomains were categorized into low, moderate, and high using tertile calculation (splitting data into three equal groups). Multiple linear regression models with a forward stepwise method were fit to determine significant predictors of the accessibility to healthcare services. The level of significance was set at *p* < 0.05.

## 4. Results

A total of 240 Myanmar migrant workers were included in this study.  Table 1 provides detailed information on characteristics for the migrants. The majority were young adults aged 25 to 34 years. The ratio of men and women was nearly equally distributed and most were married. Around 78% had below high school level of education and 82.1% had a monthly income of at least 8,000 bahts. Almost half of the subjects stated that they had health problems in the previous year.

Table 2 shows the distribution of the policy literacy scores. Based on the overall total scores, the majority of participants (36.3%) showed a high level of policy literacy, while 30.8% and 32.9% had a low and moderate level, respectively. Similarly, the majority of participants had high levels in all subdomains of policy literacy.

Table 3 shows the distribution of the scores concerning barriers to getting care. The same number of participants (34.2%) experienced barriers to getting care at both moderate and high levels, whereas 31.6% experienced a low level. Similarly, the majority of participants had high levels in all subdomains of barriers to get care, except in financial barriers and health system barriers where the majority had a moderate level of 41.2% and 36.3%, respectively.

Table 4 shows the distribution of the scores concerning the accessibility to healthcare services. Based on the total scores, the majority of participants (35.8%) had a high level, while 31.3% and 32.9% had a low and moderate level, respectively. Similarly, the majority of participants had a high level in all domains of accessibility to healthcare services.

Table 5 shows the results of the correlation analysis among the accessibility to healthcare services of Myanmar migrant workers and correlated factors such as policy literacy, barriers to get care, and certain individual characteristics. A significant positive correlation was found between policy literacy and the accessibility to healthcare services, whereas a significant negative correlation was found between barriers to care and the accessibility to healthcare services. A significant correlation between the ability to get the care and individual characteristics such as gender, education, and health status was also observed. Education and health status were positively but weakly correlated with the accessibility to healthcare services. However, gender was negatively and moderately correlated with the accessibility to healthcare services.

Table 6 shows the results of the multiple linear regression analysis to identify the significant predictors of the accessibility to healthcare services. The assumption for normality, linearity, and homoscedasticity was met. No multicollinearity was observed among the variables. The initial model that included only policy literacy as an independent variable explained 31.1% of the variance of the accessibility to healthcare services. However, independent variables of policy literacy, barriers to getting care, and gender were retained in the best predictive model for the accessibility to healthcare services which showed 43.2% of the total variance (β = 0.351, β = −0.325, and β = 0.194 respectively, *p* < 0.001). Therefore, Myanmar migrant workers with high policy literacy, low level of barriers, and female migrant workers had high accessibility to healthcare. A comparison of the two models is shown in Table 7.

## 5. Discussion

In the current study, the majority of participants were young adults (mean age = 31.6 years). This is consistent with previous studies performed in Thailand [9, 23]. Young people are more likely than older people to migrate as they are in the working-age group and also more active [3]. Also similar to another study [24], 97% of the subjects were Buddhists which is not surprising as most citizens of Myanmar are Buddhists [25]. Additionally, since most participants were married, the majority (81%) thus lived with family members. The majority of the migrant workers (82.1%) had a monthly income of at least 8,000 bahts while the average monthly income/household of Thai citizens was 26,018 bahts. Therefore, the Myanmar migrant workers' income can be considered as very low. However, the lowest average monthly income/household in Myanmar is only 175,000 kyats, equivalent to 4,166 baht [26]. Moreover, a significant proportion of the population in Myanmar (60.8%) lives below the poverty line (using the international poverty line, USD 5.50 per day) [26]. These reasons might have contributed to the increasing number of Myanmar migrant workers in Thailand.

Most migrants perceived high levels in overall policy literacy. This result was consistent with other studies conducted among migrant workers in the northeast of Thailand [9]. The study revealed that the majority of the migrants (80.2%) knew Thai health insurance. Another study from the Trat Province of Thailand found that 48.5% of Myanmar migrant workers knew about the Compulsory Migrant Health Insurance scheme [8]. Since most of the participants from the aforementioned studies lived with family members or friends, they were able to obtain more information than those who lived alone. This type of social support can improve the ability to acquire and understand the information and to negotiate the healthcare system. Besides, the participants' young age might contribute to the majority of them having a moderate level of policy literacy regarding the CMHI scheme. Another study revealed that age is strongly associated with health literacy [27]. In this study, the result showed that almost half of the participants were between the ages of 25 and 34 years. A previous study from the United States found that adults between the ages of 25 and 39 had a higher average literacy than adults in other age groups [27].

The findings on moderate and high levels in overall barriers to getting care among majority of migrants reflect limited access to healthcare services. Migrant populations, especially those working in low-skilled sectors, encounter barriers to get care at all three levels (policy arena, healthcare system, and individual level) [14]. This may be explained by the fact that more than half of the participants in this study reported that long wait time discouraged them from seeking care, a result that is consistent with previous studies [15, 20]. Besides, lack of proficiency in the local language or Thai, discrimination by local people, and lack of social network might prohibit their utilization of health services.

The majority of participants had a high level of accessibility to healthcare services. This can be influenced by the marital status of migrant workers. Most of the participants were married thus able to get support from their partners to access the health services. A systematic review using Andersen's behavioral model of healthcare utilization revealed that married people use health services more than single people [28]. Besides, age was found to be one of the predisposing factors in accessing health services [28]. The majority of participants were between the ages of 17 and 44. This is consistent with a previous study which revealed that young women aged 18 to 44 were more likely to see a doctor than their older counterpart [29].

This study's finding which indicated that policy literacy was the strongest predictor of accessibility to healthcare services suggests that migrant workers with higher policy literacy level are more likely to access healthcare. Such finding supports previous studies on determinants of access to healthcare in which literacy was one of the demand-side determinants that helped to increase the ability to perceive the need for care [30]. Similarly, the result is also congruent with a previous study which revealed that individuals with low health literacy were significantly more likely to delay seeking healthcare [13]. Therefore, policymakers and healthcare providers must offer adequate information about the healthcare programs within the Compulsory Migrant Health Insurance scheme to the migrant worker population. Using social media may be one way of information sharing in addition to other methods.

Moreover, barriers to getting care were found to be negatively associated with the accessibility to healthcare services. The participants who had low barriers to getting care were more likely to access healthcare, a result that is consistent with previous studies [16, 18, 24]. The ability to access health services by the migrant workers was mainly affected by language barriers, healthcare costs, and location of services. Therefore, healthcare providers should consider the economic background and working locations of migrant workers to help reduce their barriers to get care. For the language barrier, hiring health volunteers or interpreters who can speak both Thai and Burmese at local health facilities should be considered as part of the public health intervention and policies.

Among the selected variables for personal factors, gender was found to be the only significant predictor of the accessibility to healthcare services. According to Aday and Andersen (1998), gender is one of the predisposing factors in accessing healthcare [31]. In this study, female migrants achieved a higher accessibility to healthcare services than their male counterparts, a result that is consistent with two previous studies [32, 33]. As in other Asian countries, males are considered as the leader of the family in Myanmar culture. Consequently, the superior position in the family prohibited males to achieve proper healthcare as they were too busy to go to the hospital.

Finally, this study highlights the need for increasing migrant workers' perceived level of information about Thai health insurance and reducing their barriers to getting care. Information on healthcare programs should be proclaimed clearly by the policymakers and healthcare providers to the migrants. Besides, healthcare providers must establish an understanding of the migrant patient's culture, beliefs, and economic background.

### 5.1. Limitations

This study presented some limitations. First, interviewing participants who were unable to read or write the Myanmar language may have caused social desirability bias. Second, selection bias may occur due to the sampling method, thus the results may not be considered representative of Myanmar migrant workers in general. Third, the study being conducted only in Songkhla Province, which is located in Southern Thailand, may limit the generalizability of the findings to the population of Myanmar migrants in Thailand as a whole. Finally, a cross-sectional study on causal relationships in migrant workers' perceived level of policy literacy, barriers to getting care, and the accessibility to healthcare services may present inconclusive findings.

## 6. Conclusion

The findings of this study provide information about the ability to get the care and its determinants among Myanmar migrant workers in Songkhla Province of Thailand. The results indicate that policy literacy, barriers to get care, and gender can significantly predict the accessibility to healthcare services. Therefore, to improve the overall accessibility to healthcare services among the migrant population, healthcare programs should include strategies to maximize the distribution of information about the Compulsory Migrant Health Insurance scheme while minimizing the barriers to get care. Public health policymakers and government officials should cooperate with healthcare providers and employers of migrant workers to provide effective and better healthcare services for migrant workers. Besides, information about the Compulsory Migrant Health Insurance scheme should be made available in Burmese language and displayed at the hospitals and other places where migrant workers can easily access. It is recommended that future studies should explore more influencing factors on the accessibility to healthcare services while also adopt a random sampling method to improve the generalization of the findings. Furthermore, it is recommended that qualitative studies using in-depth interviews and focus group discussions will provide essential information for policymakers and healthcare providers to develop effective healthcare programs for the migrant population.

## Figures and Tables

**Table 1:** Demographic characteristics of Myanmar migrant workers (*N* = 240).

Personal characteristics	Frequency	Percentage
Age (years) (Mean = 31.65, SD = 8.05)		
17–24	47	19.6
25–34	115	47.9
35–44	58	24.2
45–54	18	7.5
55–60	2	0.8

Gender		
Male	117	48.8
Female	123	51.2

Religion		
Buddhist	233	97.1
Christian	1	0.4
Muslim	6	2.5

Educational level		
High school and above	26	10.8
Below high school	187	77.9
^*∗*^Monastic education	6	2.5
No education	21	8.8

Marital status		
Single	50	20.8
Married	188	78.2
Widowed/divorced/separated	2	0.8

Occupational group		
Agriculture	60	25
Construction	66	27.5
Seafood processing	54	22.5
Services (shop/restaurant)	60	25

Monthly income (Median = 9,000, IQR = 8,000–10,000)		
3,000–8,000	43	17.9
8,001–9,600	131	54.6
9,601–20,000	66	27.5

Job satisfaction		
Yes	209	87.1
No	31	12.9

Personal characteristics	Frequency	Percentage
Duration of stay in Thailand (years)		
(Mean = 8.27, SD = 5.45)				
1–5	83	34.6
6–10	110	45.8
10–30	47	19.6

Health status within the past year		
Sick	112	46.7
Healthy	128	53.3

Living status		
Alone	20	8.3
With family	195	81.3
With friends	25	10.4

Experience using CMHI		
Yes	127	52.9
No	113	47.1

CMHI, Compulsory Migrant Health Insurance. ^*∗*^Monastic education is considered informal education and often accessed by children from very poor families or from remote locations and ethnic minorities.

**Table 2:** Policy literacy regarding Compulsory Migrant Health Insurance of Myanmar migrant workers (*N* = 240).

Policy literacy	Frequency	Percentage
Total		
Low (≤82.00)	74	30.8
Moderate (82.01–96.00)	79	32.9
High (>96.00)	87	36.3

Ability to find		
Low (≤21.00)	80	33.3
Moderate (21.01–24.00)	66	27.5
High (>24.00)	94	39.2

Ability to understand		
Low (≤14.00)	72	30.0
Moderate (14.01–16.00)	55	22.9
High (>16.00)	113	47.1

Ability to appraise		
Low (≤26.00)	62	25.8
Moderate (26.01–31.00)	87	36.3
High (>31.00)	91	37.9

Ability to communicate		
Low (≤18.00)	80	33.3
Moderate (18.01–22.00)	76	31.7
High (>22.00)	84	35.0

**Table 3:** Barriers to getting care regarding Compulsory Migrant Health Insurance of Myanmar migrant workers (*N* = 240).

Barriers to get care	Frequency	Percentage
Total		
Low (≤48.00)	76	31.6
Moderate (48.01–60)	82	34.2
High (>60.00)	82	34.2

Personal barriers		
Low (≤11.00)	61	25.4
Moderate (11.01–14.00)	80	33.3
High (>14.00)	99	41.3

Financial barriers		
Low (≤5.00)	54	22.5
Moderate (5.01–8.00)	99	41.2
High (>8.00)	87	36.3

Social barriers		
Low (≤7.00)	56	23.3
Moderate (7.01–9.00)	79	32.9
High (>9.00)	105	43.8

Healthcare provider barriers		
Low (≤5.00)	66	27.5
Moderate (5.01–7.00)	52	21.7
High >7.00)	122	50.8

Health system barriers		
Low (≤10.00)	73	30.4
Moderate (10.01–13.00)	87	36.3
High (>13.00)	80	33.3

Work situation barriers		
Low (≤3.00)	43	17.9
Moderate (3.01–5.00)	78	32.5
High (>5.00)	119	49.6

**Table 4:** Accessibility to healthcare services regarding Compulsory Migrant Health Insurance of Myanmar migrant workers (*N* = 240).

Accessibility to healthcare services	Frequency	Percentage
Total		
Low (≤65.00)	75	31.3
Moderate (65.01–72.00)	79	32.9
High (>72.00)	86	35.8

Geographic accessibility		
Low (≤4.00)	55	22.9
Moderate (4.01–4.99)	39	16.3
High (>4.99)	146	60.8

Availability		
Low (≤24.00)	72	30.0
Moderate (24.01–27.00)	81	33.7
High (>27.00)	87	36.3

Financial accessibility		
Low (≤17.00)	71	29.6
Moderate (17.01–19.00)	79	32.9
High (>19.00)	90	37.5

Acceptability		
Low (≤17.00)	67	27.9
Moderate (17.01–17.99)	42	17.5
High (>17.99)	131	54.6

**Table 5:** Correlations among individual characteristics, policy literacy, barriers to getting care, and accessibility to healthcare services of Myanmar migrant workers (*N* = 240).

	Gender	Education	Marital status	Income	Health status	Policy literacy	Barriers to get care
Gender^a^							
Education	−0.072^*∗*^						
Marital status	−0.013	0.053					
Income	0.278^*∗∗*^	0.017	0.056				
Health status	0.023^*∗*^	−0.004	0.208^*∗*^	−0.048			
Policy literacy	−0.154^*∗∗*^	0.021	0.195^*∗*^	0.281^*∗∗*^	0.140^*∗*^		
Barriers to get care	0.166^*∗∗*^	−0.006	0.063	−0.162^*∗*^	−0.117^*∗*^	−0.544^*∗∗*^	
Accessibility to healthcare services	−0.301^*∗∗*^	0.107^*∗*^	0.095	0.021	0.173^*∗*^	0.557^*∗∗*^	−0.548^*∗∗*^

^*∗*^ *p* < 0.05; ^*∗∗*^*p* < 0.001. ^a^Dummy coded variable (0 = male, 1 = female).

**Table 6:** Multiple regression analysis of policy literacy, barriers to getting care, and gender on accessibility to healthcare services by Myanmar migrant workers (*N* = 240).

Best model	B^a^	SE^b^	*β* ^c^	*t*	*p* value
Policy literacy	0.211	0.035	0.351	5.986	<0.001
Barrier to get care	−0.212	0.038	−0.325	−5.526	<0.001
Gender^d^	3.161	0.815	0.194	3.880	<0.001

^a^Unstandardized regression coefficients. ^b^Standard error. ^c^Standardized regression coefficients. ^d^Dummy coded variable (0 = male, 1 = female).

**Table 7:** The coefficients of determination in the initial and best predictive models for the accessibility to healthcare services (*N* = 240).

Model	*R*	*R* ^2^	*R* ^2^ change	Residual statistics	
				*F*	*p* value
Initial^a^	0.557	0.331	0.331	107.243	<0.001
Best^b^	0.657	0.432	0.036	59.787	<0.001

Dependent variable: accessibility to healthcare services. ^a^Predictor: policy literacy. ^b^Predictors: gender, barriers to get care, and policy literacy.

## Data Availability

The data used to support the findings of this study are available from the corresponding author upon request.
